# Nanocomposite Furcellaran Films—the Influence of Nanofillers on Functional Properties of Furcellaran Films and Effect on Linseed Oil Preservation

**DOI:** 10.3390/polym11122046

**Published:** 2019-12-09

**Authors:** Ewelina Jamróz, Pavel Kopel, Joanna Tkaczewska, Dani Dordevic, Simona Jancikova, Piotr Kulawik, Vedran Milosavljevic, Kristyna Dolezelikova, Kristyna Smerkova, Pavel Svec, Vojtech Adam

**Affiliations:** 1 Department of Chemistry, University of Agriculture, Balicka Street 122, PL-30-149 Cracow, Poland; ewelina.jamroz@urk.edu.pl; 2 Department of Inorganic Chemistry, Faculty of Science, Palacky University, 17. listopadu 12, CZ-771 46 Olomouc, Czech Republic; 3 Faculty of Electrical Engineering and Communication, Department of Microelectronics, Brno University of Technology, Technicka 3058/10, CZ-616 00 Brno, Czech Republic; 4 Department of Animal Product Technology, Faculty of Food Technology, University of Agriculture in Cracow, Balicka 122 Street, PL-30-149 Cracow, Poland; tkaczewska@gmail.com (J.T.); kulawik.piotr@gmail.com (P.K.); 5 Department of Vegetable Foodstuffs Hygiene and Technology, Faculty of Veterinary Hygiene and Ecology, University of Veterinary and Pharmaceutical Sciences Brno, Palackeho tr. 1946/1, CZ-612 42 Brno, Czech Republic; dani_dordevic@yahoo.com (D.D.); JANCIKOVAS@vfu.cz (S.J.); 6 Department of Technology and Organization of Public Catering, South Ural State University, Lenin Prospect 76, 454080 Chelyabinsk, Russia; 7 Department of Chemistry and Biochemistry, Faculty of AgriSciences, Mendel University in Brno, Zemedelska 1, CZ-613-00 Brno, Czech Republic; grizlidripac@gmail.com (V.M.); kristyna.dolezelikova@mendelu.cz (K.D.); kristyna.smerkova@ceitec.vutbr.cz (K.S.); pavel.svec@mendelu.cz (P.S.); vojtech.adam@mendelu.cz (V.A.); 8 Central European Institute of Technology, Brno University of Technology, Purkynova 123, CZ-612 00 Brno, Czech Republic

**Keywords:** furcellaran, graphene oxide, maghemite nanoparticles, carbon quantum dots, nanocomposite films, active properties, linseed oil preservation

## Abstract

Nanocomposite films that were based on furcellaran (FUR) and nanofillers (carbon quantum dots (CQDs), maghemite nanoparticles (MAN), and graphene oxide (GO)) were obtained by the casting method. The microstructure, as well as the structural, physical, mechanical, antimicrobial, and antioxidant properties of the films was investigated. The incorporation of MAN and GO remarkably increased the tensile strength of furcellaran films. However, the water content, solubility, and elongation at break were significantly reduced by the addition of the nanofillers. Moreover, furcellaran films containing the nanofillers exhibited potent free radical scavenging ability. FUR films with CQDs showed an inhibitory effect on the growth of *Staphylococcus aureus* and *Escherichia coli*. The nanocomposite films were used to cover transparent glass containers to study the potential UV-blocking properties in an oil oxidation test and compare with tinted glass. The samples were irradiated for 30 min. with UV-B and then analyzed for oxidation markers (peroxide value, free fatty acids, malondialdehyde content, and degradation of carotenoids). The test showed that covering the transparent glass with MAN films was as effective in inhibiting the oxidation as the use of tinted glass, while the GO and CQDs films did not inhibit oxidation. It can be concluded that the active nanocomposite films can be used as a desirable material for food packaging.

## 1. Introduction

The use of biopolymer films as an alternative to synthetic packaging is becoming increasingly attractive in the food packaging industry, as it can help to mitigate the effects of environmental pollution generated by plastic materials. Proteins and polysaccharides are often used as raw materials for obtaining biopolymer films [1,2,3,4,5]. One such polysaccharide with gel-forming properties is furcellaran, which is obtained from red algae *Furcellaria lumbricalis*. This negatively charged polysaccharide consists of a structurally repeating unit of alternating 3-d-galactopyranose and 4-linked α-d-galactopyranose residues, and the last residues can occur as 3,6-anhydro, which can be sulfated at position 2 [6]. Furcellaran can be used as the matrix of many biopolymer films due to their film forming ability [7,8,9]. However, like all biopolymer films, it has its disadvantages, which include high gas permeability and poor mechanical properties. Nanoparticles [10] and plant extracts [11] are added to improve these properties.

Nanoparticles of biodegradable polymers have aroused continuous interest as, for example, a drug delivery system in the past [12,13]. The massive progress in nanotechnology has allowed to produce advanced materials with very good biological, magnetic, optical, and mechanical properties. Nanotechnology is now focusing increasing attention on obtaining organic/inorganic nanocomposites to obtain materials with new hybrid properties. One promising nanocomposite material is nanocomposite films, which consist of at least two components—polymers, as the film-forming matrix (continuous phase) and nanostructured components of nanometric size, often acting as a nanofiller (discontinuous phase) [14]. In the literature, there are many works researching the effect of the addition of nanofillers (nanoclays, nanoparticles, etc.) on the properties of biopolymer films [15,16,17,18].

Magnetic nanoparticles are suitable for the production of functional nanostructures due to their physical properties, primarily their size and shape. Iron oxide magnetite (Fe_3_O_4_) and its oxidized form, maghemite (γ-Fe_2_O_3_), are the most commonly used magnetic nanoparticles in medicine and biotechnology. The most important properties of this type of nanoparticles include controlled shape, non-toxicity, biocompatibility, large surface area, high crystallinity, and the ability to be well dispersed in an aqueous environment [19].

Carbon quantum dots (CQDs) are a new group of nanomaterials that are smaller than 10 nm with interesting physicochemical properties, such as low toxicity, high solubility in water and other solvents, stable photoluminescence, and flexibility of surface modification [20]. Graphene oxide (GO) is a single-layer nanomaterial with the high surface area, prepared from natural graphite and it has high oxygen content. It has many rich oxygen-containing functional groups (e.g., hydroxyl, epoxide, and carboxyl) in its structure that can interact with the biopolymer matrix [21,22]. The addition of GO increases the mechanical strength and the moisture barrier of the film [23,24].

The oxidation of oils is the main cause of formation of off-odors and off-tastes within them, resulting in their reduced quality, rancidity, and compromised safety. The oxidation rate depends on the presence of oxidation promoters, such as metal ions, light, increased temperature, or oxidative enzymes. [25]. Out of these, light exposure is the most important factor that causes oxidation of shelf stored oils. This is especially important when storing cold-pressed oils, which are rich in polyunsaturated fatty acids [26]. Therefore, it is important to protect stored oils from light exposure to extend their shelf-life. One possible method is the use of packaging that is coated with environmentally friendly, biodegradable polymer films with UV-barrier properties.

The aim of the study was to develop new furcellaran films while using different nanofillers: maghemite nanoparticles (MAN), graphene oxide (GO), and carbon quantum dots (CQDs), and to evaluate their microstructure, physical, mechanical, and biological (antioxidant and antimicrobial) properties. To the best of our knowledge, there have been no other studies reporting on obtaining nanocomposite films with the presented compounds. The secondary aim was to establish the possible application of the newly developed films as UV-barrier coatings for preventing the oxidation of stored cold-pressed oil after exposure to UV-B radiation.

## 2. Materials and Methods

### 2.1. Materials

Furcellaran (M_w_ 2.951 × 10^5^ and chemical content: carbohydrates 79.61%; protein 1.18%; and, fat 0.24%) was obtained from Est-Agar AS (Karla village, Estonia). Cold pressed flaxseed oil (Natur Farm, Galanta, Slovakia) was purchased from a local shop in Brno, Czech Republic. The oil was sealed in 250 mL dark tinted bottles. 

All the solvents were procured from Sigma-Aldrich (St. Louis, MO, USA).

### 2.2. Methods

#### 2.2.1. Synthesis of Graphene Oxide (GO)

GO was prepared while using the modified Hummers method by oxidation of graphite flakes [27,28,29]. The graphite flakes (2 g), sodium nitrate (1 g), and potassium permanganate (6 g) were supplemented to stirred cooled concentrated sulfuric acid (46 mL). The blend was stirred overnight. Water (400 mL) was added slowly and the blend was heated. After cooling, hydrogen peroxide solution was added until the color turned yellow. The decantation was applied and water was changed several times. GO was used as a water suspension. The hydrodynamic diameter of nanoparticles was checked by the dynamic light scattering (DLS) technique on a Malvern Zetasizer (NANO-ZS, Malvern Instruments Ltd., Worchestershire, United Kingdom). The hydrodynamic diameters were 848 ± 290 nm.

#### 2.2.2. Preparation of Carbon Dots (CQDs)

Citric acid monohydrate (2.1 g) was dissolved in 45 mL of MilliQ water and 3,3’-diamino-N-methyl-dipropylamine (1.7 mL) was slowly added to the solution while stirring. Finally, water was added to reach 50 mL. Each 2 mL of the solution was heated (300 W, 130 °C, 10 min.) in a glass vial in a Multiwave 300 microwave oven (Anton Paar, Graz, Austria). The solutions were dialysed against water for 24 h while using the maxi D-Tube dialyzer that was supplied by Millipore (EMD, San Diego, CA, USA). The hydrodynamic diameters were 2.6 ± 0.6 nm.

#### 2.2.3. Preparation of Modified Maghemite (MAN)

Nanomaghemite was prepared, as previously reported [30,31]. Fe(NO_3_)_3_·9H_2_O (1.5 g) was dissolved in water (80 mL) and a solution of NaBH_4_ (0.2 g) dissolved in 10 mL of 3.5% NH_3_ was added under stirring, and then heated for 2 h at 100 °C. After cooling, the mixture was left overnight and maghemite was separated by a magnet, washed several times with water, and dispersed in methanol (100 mL). Tetraethyl orthosilicate (200 µL) was poured inside and the mixture was shaken for 2 h, followed by the addition of 3-(triethoxysilyl)propyl isocyanate (200 µL). After 2 h, 1 mL of 25% ammonia solution was added. The solution was left to stir (3 h) and water was added (50 mL). The mixture was stirred overnight, separated by a magnet, washed several times with water, and then left in water (50 mL). The hydrodynamic diameters were 163 ± 40 nm.

#### 2.2.4. Preparation of Nanocomposite Furcellaran Films 

Furcellaran films and nanocomposite films were obtained by the solution casting method. Furcellaran film was prepared by dissolving 0.5 g of furcellaran powder in 50 mL of distilled water and stirred for an hour on a magnetic stirrer (300 rpm). Subsequently, 0.25 mL of glycerol was added and mixed for half an hour. The film-forming solution that was prepared in this way was referred to as the control sample (furcellaran (FUR)). Nanofillers (GO, CQDs, and MAN) were added to each film-forming solution to prepare nanocomposite films. The content of nanofillers in composite films was 2.5 wt.%. The final concentration of nanofillers was carried out according to preliminary experiments (data not shown). Each type of prepared film-forming solution was poured into petri dishes (90 mm dimeter) and then dried (23 ± 1 °C) under a fume hood for 24 h.

#### 2.2.5. Fourier Transform Infrared Spectra (FTIR) 

FTIR analysis of FUR and FUR nanocomposite films was measured while using a Fourier-transform infrared spectrophotometer (MATTSON 3000 FT-IR, Madison, WI, USA), in the wavelength range of 4000–400 cm^−1^ with 4 cm^−1^ resolution.

#### 2.2.6. X-ray Diffraction (XRD)

XRD patterns of nanocomposite films were measured by using X’Pert PRO (PANalytical B.V., EA Almelo, Netherlands) diffractometer operated at 40 kV and 30 mA by using Ni-filtered Cu Kα radiation. All the patterns were recorded in the range of 5–72°, with a step size of 0.05°. The samples were prepared as oriented thin films by drying a suspension of polymer composite on a glass slide.

#### 2.2.7. Scanning Electron Microscopy (SEM)

For FUR: The morphology and composition were examined by scanning emission microscopy while using a Tescan MIRA 2 that was equipped with a field emission gun (Tescan Ltd., Brno, Czech Republic). The SEM was fitted with an external SE detector with working distance that was set between 14.95–15.47 mm and at 15 kV acceleration voltage. Pixel images (768 × 858) were obtained at 1000 folds magnification covering a sample area of 216.7–217.4 µm.

For other samples: The morphology and composition were examined by scanning emission microscopy on a Tescan MAIA 3 that was equipped with a field emission gun (Tescan Ltd., Brno, Czech Republic). Best images were obtained while using the external SE detector at working distance between 6.81–10.99 mm and at 5 kV acceleration voltage. The pixel images (768 × 858) were obtained at 1000 folds magnification covering a sample area of 208 µm. Full frame capture was performed in analytical mode and accumulation of image with image shift correction enabled, and it took about 1.5 min. with the ~1 µs/pixel dwell time. Spot size was set at 17 nm.

#### 2.2.8. Physical Properties

##### Thickness

The film thickness was measured while using a micrometer Mitotuyo, no. 7327 (Kawasaki, Japan). Ten readings were taken along the perimeter of the film with the result reported as the mean value.

##### Mechanical Properties

Tensile strength (TS) and elongation at break (EAB) were measured while using a TAxT2i (Stable Micro System, Surrey, UK) texturometer. The initial distribution of adhesion was set at 50 mm and the deformation rate was set at 5 mm/min.

##### Water Content and Solubility

Water content and solubility of the films was determined according to Souza, et al. [32]. 

The samples of the films (3 × 3 cm) were weighed and dried in an oven (105 °C) for 3 h to determine water content. The dried films were weighed again, and the water content was calculated according to the following equation:

(1) $$\mathrm{Water}\;\mathrm{content}\;\left[\%\right]=\left(M0-M1\right)/M1\times 100$$

where M0 is the initial dry weight of films (under a fume hood for 24 h) and M1 is the final dry weight of films (105 °C in oven).

The samples of the films (3 × 3 cm) were weighed and immersed in distilled water for 24 h to determine solubility. The undissolved parts of films were dried in an oven (105 °C) for 3 h and weighed again. The solubility of the film was calculated according to the following equation:

(2) $$\mathrm{Solubility}\;\left[\%\right]=\left(M0-M1\right)/M0\times 100$$

where M0 is the initial dry weight of films (under a fume hood for 24 h) and M1 is the final dry weight of films (105 °C in oven).

##### UV-Visible Absorption Spectra

The UV-Vis spectra of every type of film were recorded from 200 to 800 nm while using a UV-5500 spectrophotometer (Metash, Shanghai, China).

##### The Color Measurement of Films

L* (lightness), a* (redness), and b* (yellowness) parameters were determined according to Mehdizadeh, Tajik, Razavi Rohani, and Oromiehie [33] while using a CR 200 Minolta Chromameter (Osaka, Japan). Each sample of nanocomposite films was determined using three independent repetitions with five measurements taken on each repetition (n = 3 × 5), with a standard white plate used as background. The total color difference (ΔE) was calculated according to literature [33].

#### 2.2.9. Biological Properties of Films

##### Antibacterial Properties of Films

The antibacterial properties of tested films were performed by the disc diffusion method on *Staphylococcus aureus* (CCM 4223), *Escherichia coli* (CCM 7929), and *Salmonella enterica* (CCM 7189) bacterial strains. The bacterial strains were cultured on 5% Sheep Blood agar (SBA) overnight and then diluted in saline to OD600 = 0.1 AU. Petri dishes with Mueller–Hinton agar were covered by diluted strains while using a swab and the films were laid on top of the agar. Prepared Petri dishes were incubated overnight at 37 °C and inhibition of bacterial growth was then evaluated.

##### Antioxidant Properties of Films

The films were ground and homogenised while using a laboratory mill. A film solution was prepared by dissolving 375 mg of the ground film in 50 mL of distilled water. The solution was heated to 40 °C until the film completely dissolved. The FRAP, DPPH, and metal chelating ability methods were used to measure the antioxidant properties of the films.

The FRAP method was performed according to Behbahani*,* et al. [34] with modifications. The film solution (0.4 mL) was mixed with 3.6 mL of FRAP reagent prepared from 300 mM/L acetic buffer, 10 mM/L of TPTZ, and 20 mM/L of FeCl_3_·3H_2_O (10:1:1). The sample prepared in such a manner was incubated in the dark at 37 °C for 15 min. Subsequently, absorbance was measured at 595 nm while using a Helios Gamma spectrophotometer (Thermo Fisher Scientific, Waltham, MA, USA). The results were calculated as the amount of mM FeSO_4_/L, based on a standard curve of FeSO_4_·7H_2_O, which relates the concentration of FeSO_4_·7H_2_O (mM) to the absorbance at 595 nm.

The DPPH method is a measure of radical scavenging activity and it was performed according to Adilah*,* et al. [35]. 2.8 mL of film extract was mixed with 0.2 mL of 0.1 mM solution of 2,2-diphenyl-1-picrylhydrazyl in ethanol. The mixture was incubated in the dark for 10 min. After this, absorbance was measured at 517 nm while using a spectrophotometer (Thermo Fisher Scientific, Waltham, MA, USA). DPPH scavenging activity was calculated according to the equation presented below:

(3) $$\mathrm{DPPH}\;\mathrm{scavenging}\;\mathrm{activity}\;\left[\%\right]=\left[\left(\mathrm{Abs}\;\mathrm{DPPH}-\mathrm{Abs}\;\mathrm{sample}\right)/\mathrm{Abs}\;\mathrm{DPPH}\right]\times 100$$

The metal chelating activity in the samples was determined using the method that was described by Carter [36] with some modifications. A mixture containing 1 mL of film extract, 3.7 mL of distilled water, and 0.1 mL of 2 mM ferrous chloride (FeCl_2_) was created. After 3 min., 0.2 mL of 5 mM ferrozine was added to inhibit the reaction. The sample was vortexed and then incubated at room temperature for 10 min. Absorbance was measured at a wavelength of 562 nm while using a spectrophotometer (Thermo Scientific, Waltham, MA, USA). Water was used as a blank sample, and the control was a reagent test. Chelating activity was calculated while using the following equation:

(4) $$\mathrm{Fe}\;\mathrm{chelating}\;\left[\%\right]=\left(1-A_{P}/A_{0}\right)\times 100$$

#### 2.2.10. Oil Stability after UV Treatment

##### UV-B Treatment

Five mL of cold pressed flaxseed oil was placed on round Petri dishes (30 mm diameter) that were made from transparent glass or into amber tinted bottles (30 mm diameter). The Petri dishes were either left uncovered (UV-Control) or covered completely with films of either FUR + MAN, FUR + GO or FUR + CQDs. Afterwards, the Petri dishes and amber tinted bottles with oil were placed in a dark chamber on a TCP-26.LMX Vilber Lourmat UV-Transilluminator (Eberhardzell, Germany) equipped with 6 × 8 W UV tubes operating at 312 nm wavelength. The treatment was carried out for 30 min. After the treatment, the oils were collected in amber tinted glass bottles and stored in the dark. The samples were subjected to analyses to measure the UV-induced changes and oxidation: peroxide value (PV), free fatty acids (FFA) malondialdehyde (MDA) content, carotenoids content, and FRAP antioxidant power after one day of treatment and compared with the results of untreated cold pressed flaxseed oil. The treatment and all analyses were performed while using three independent repetitions.

##### PV, FFA, and MDA Content

The peroxide value was determined based on the ISO 3960:2017 standard. Five g of oil mixed with 30 mL of glacial acetic acid and chloroform mixture (3:2 ratio) was vortexed for 60 sec and then mixed with 30 mL of distilled water and 5 mL of 1% starch solution. The sample was titrated with 0.01M Na_2_S_2_O_3_. 

Free fatty acids were measured by the acid value method according to ISO 660:2009 standard. Five g of oil was mixed with 50 mL diethylether. After addition of indicator (1 mL of phenolphthalein) the mixture was vortexed for 1 min. and titrated with 0.1 M KOH. 

The carotenoids content was analyzed by measuring the absorbance of oil at 383 nm while using a CECIL CE 7210 spectrophotometer (Cecil Instruments, Milton, UK) with cyclohexane used as a blank.

To measure MDA content, 3 mL of oil was mixed with 6 mL of 20% TCA and incubated in a dark water bath at 95 °C for 20 min. After cooling in an ice water bath the samples were centrifuged at 5100 rpm for 15 min. and 2 mL of supernatant was mixed with thiobarbituric acid solution (167 mg TBA in 20 mL of water). The samples were incubated in a dark water bath at 95 °C for 60 min. and then centrifuged at 5100 rpm for 15 min. Subsequently, 850 µL of the bottom layer was collected and filtered through 0.45 µm filters into amber tint chromatography vials and then subjected to HPLC analysis. The analysis was performed on an Agilent Infinity 1260 (Santa Clara, CA, USA) that was equipped with a DAD detector. The separation was performed according to the method that was described by Konieczka, et al. [37]. HPLC grade water (A) and acetonitrile (B) were used as mobile phases while using the following protocol: 90% A and 10% B for 10 min., 65% A and 35% B from 10 to 30 min., 10% A and 90% B from 30 to 31 min. The separation was performed with a Zorbax SB-C18 4.6 × 250 mm (Agilent Technologies, Santa Clara, CA, USA). The flow rate was 1.5 mL/min., the injection volume was 10 μL and the DAD setting was 53 nm. The temperature of the column was maintained at 45 °C. All the analyses were performed in triplicate. 

### 2.3. Statistical Analysis

All the analyses were performed in triplicate unless otherwise stated. The statistical analysis was performed using Statistica v 13.0 (StatSoft, Tulsa, OK, USA) software. The normality of the results was checked while using Saphiro–Wilk test, and Box-Cox transformation were used for variables with non-normal distribution. The homogeneity of variances was checked while using Levene’s test, after which the results were subjected to one-way analysis of variances. The individual differences between groups were established using Tukey test with the probability value of P < 0.05. 

## 3. Results and Discussion

### 3.1. Structural and Physical Properties

**Figure 1:**
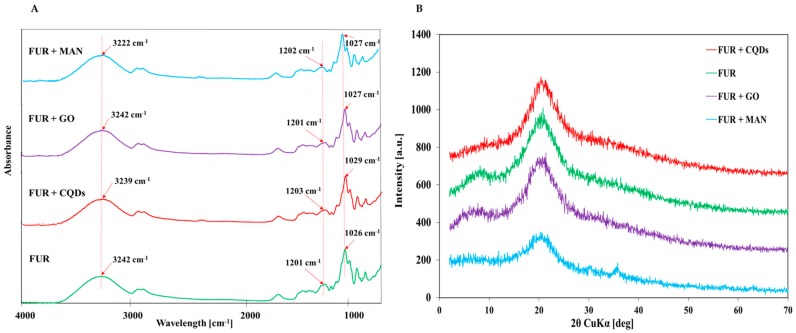
(**A**) Fourier Transform Infrared Spectra (FTIR) analysis (**B**) X-ray diffraction (XRD) analysis of furcellaran films and nanocomposite films.

Figure 1A shows the FTIR spectra of FUR, FUR + MAN, FUR + GO, FUR + CQDs films. In the FTIR spectrum of furcellaran films, characteristic bands from SO groups and C–O stretch vibrations (at 1201 and 1026 cm^−1^, respectively) were observed.

No additional peaks from nanofillers in the nanocomposite film spectrum were observed. This might have been due to the low concentration of nanofillers in the film structure. In addition, nanoparticles could have been anchored in the furcellaran matrix, which could suppress the absorption band from nanofillers, which in turn could have resulted in the lack of the characteristic IR absorption of nanoparticles in composites. 

Figure 1B displayed the X-ray diffraction (XRD) pattern of FUR films and FUR nanocomposite films. In FUR films, Bragg’s reflection was observed at 2Θ = 20.1°, which indicated the crystalline nature of furcellaran [38]. All types of films were amorphous, because they exhibit an amorphous halo with a maximum at 20° 2Θ. After the addition of nanofillers, the characteristic peaks for nanocomposite films increased and they were shifted to higher angles of 2Θ = 21–22°, which might indicate a more solid crystalline structure of the nanocomposite film. The addition of GO and CQDs to FUR did not cause the presence of new peaks. The new peak in FUR + MAN films between 30° and 40° confirms the presence of γ-Fe_2_O_3_ in the furcellaran matrix [39]. 

**Figure 2:**
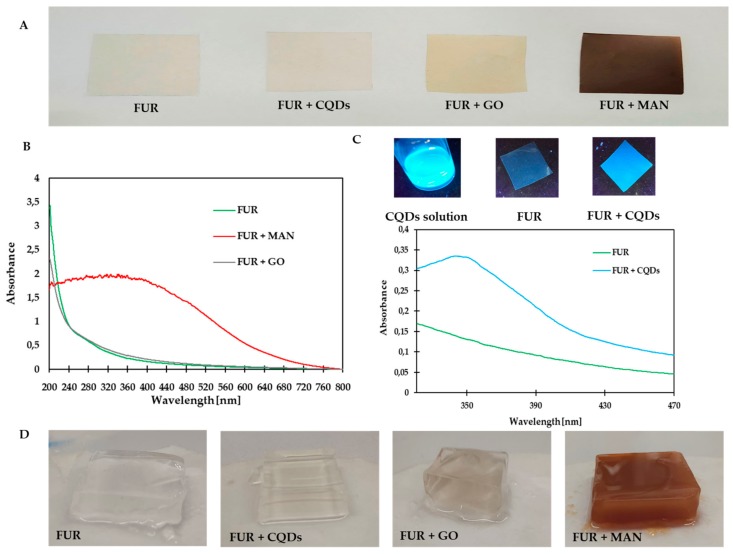
(**A**) The appearance of nanocomposite films, (**B**) UV-Vis spectrum of nanocomposite films, (**C**) UV-Vis spectrum of furcellaran (FUR) + Carbon quantum dots (CQDs) and photographs of FUR + CQDs film under UV illumination, and (**D**) appearance of film after immersion in water for 24 h.

The solvent casting method obtained nanocomposite films with a matrix of furcellaran and nanofillers (CQDs, GO, and MAN). Furcellaran films are colorless and transparent. The addition of CQDs did not change the appearance of the FUR film. Films with GO turned grey, while the FUR + MAN films were dark brown and not transparent (Figure 2A). 

Figure 2B,C show the UV-Vis absorption spectra of furcellaran films with the addition of nanofillers. Furcellaran films and FUR + GO films did not show absorption in the entire spectrum, but only within the UV radiation wavelengths. The addition of MAN to the film caused an increase in absorption, which is a convenient feature for photocatalytic applications (Figure 2B). The films with CQDs absorbed radiation in the range of 300–400 nm, which is associated with strong fluorescence (Figure 2C), may make this type of film a suitable material for sunlight blocking applications [40]. Additionally, FUR + CQDs show light blue emission under UV-Vis light (Figure 2C).

The furcellaran films formed a gel when immersed in water and it was difficult to pull them completely out of the water (Figure 2D). The addition of CQDs to the FUR films did not significantly affect the structure of the film after contact with water. However, the addition of GO and MAN to FUR films allowed for the films to be completely removed from the water. They took the form of normal films after drying.

Table 1 shows the color values of nanocomposite films. Lower L* values (lightness), higher b* values (yellowness), an a* values (redness) and ΔE (color difference) were found for films with MAN and GO as compared to FUR films. 

There were no changes in the color of the FUR film after adding CQDs. The changes resulted from the color of nanofiller solutions. The obtained color results were consistent with the appearance of the film (Figure 2A).

Table 2 presents the results regarding the effect of nanofillers on the water properties (solubility and water content) of the furcellaran films. The addition of nanofillers did not cause a decrease in the water content of furcellaran films. In the case of solubility, the addition of nanofillers affected this parameter, causing a significant decrease. The highest decrease in solubility was recorded in the FUR + MAN films. This might be due to the increased compactness of the biopolymer matrix [41]. Table 2 shows the thickness of the nanocomposite furcellaran films, the tensile strength (TS), and elongation at break (EAB) of the composite film. The addition of nanofillers did not increase the thickness of the furcellaran films. This was probably due to the low concentration of nanofillers in the matrix. The addition of MAN and GO influenced the tensile strength of FUR films. The addition of CQDs did not cause significant changes in the tensile strength parameters of the film. The increase in TS can be attributed to the good distribution of GO and MAN in the FUR matrix and the hydrogen bonds between FUR and nanofillers [1].

The elongation at the break parameter of the nanocomposite films was not significantly different from that of the furcellaran films. Usually, the addition of this type of nanofillers to biopolymer films causes a deterioration of flexibility, which is associated with a greater rigidity of films. In this case, the concentration of nanofillers might have been too low and affected this parameter. 

### 3.2. Morphology

**Figure 3:**
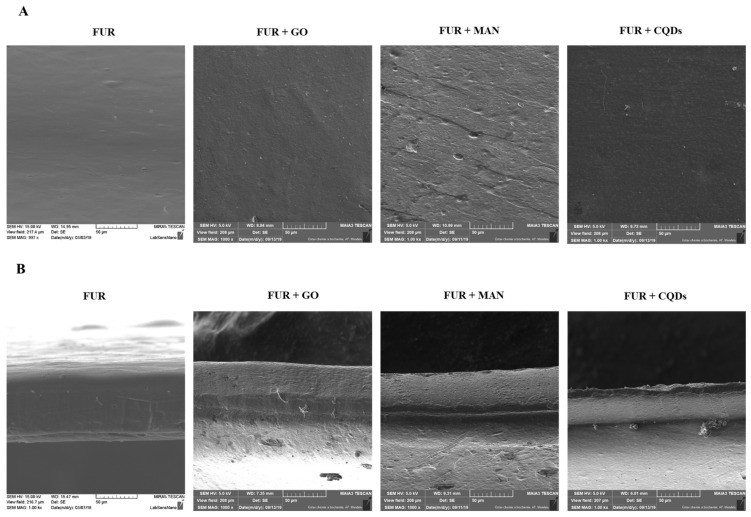
Scanning Electron Microscopy (SEM) photographs of (**A**) surface, (**B**) cross-section of furcellaran films and nanocomposite films (scale bars = 50 µm).

Scanning electron microscopy analysis was used to show the morphology on the surface and the cross section of nanocomposite films (Figure 3). The surface and the cross-sectional microstructure of the neat FUR film showed a smooth and homogenous surface. The addition of GO into the FUR film caused the presence of clusters on the surface, which showed that the GO sheets were wrapped in a FUR matrix and also adhered well to the biopolymer. For the cross-sectional microstructure, FUR + GO films showed a dense structure with clearly spaced GO sheets. The scanning microscope micrographs showed that the surface of films was relatively irregular, due to presence of maghemite nanoparticles. It should be noted that MAN tends to aggregate when mixed with the biopolymer, which is the result of their magnetic properties [42]. It has been observed that CQDs are well dispersed in a biopolymer matrix. No rough surface was observed, which might indicate the efficient filling of the biopolymer matrix.

### 3.3. Biological Properties

#### 3.3.1. Antimicrobial Properties

The antimicrobial properties of the prepared furcellaran films with the addition of different nanoparticles were evaluated against Gram-positive (*Staphylococcus aureus*) and Gram-negative bacteria (*Escherichia coli* and *Salmonella enteritidis*) via the agar disc diffusion assay (Table 3).

The diffusion abilities of the films were not observed but several foils inhibited the bacterial growth directly below them. All of the tested films indicated an inhibitory effect on *S. enteritidis* growth, except for the films containing magnetic particles. It might be assumed that this was not the effect of nanoparticle activity, but due to the activity of the matrix itself, which was furcellaran. This polysaccharide has reactive sulfate groups (SO_4_H) [43]. According to data from literature, polysaccharides that contain sulfate residue demonstrate bacteriostatic properties against *S. enteritidis* [44]. The lack of inhibitory abilities of the films with magnetic nanoparticles against the growth of *S. enteritidis* may have resulted from the fact that furcellaran might have the ability to electrostatically entrap the maghemite nanoparticles in their sulfate groups [45]. According to Yamashita, Sugita-Konishi and Shimizu [44], the removal or blockage of sulfate residues eliminate the bacteriostatic effect of polysaccharides. 

Films with the addition of CQDs showed an inhibitory effect on the increase in *S. aureus* and *E. coli*. The addition of other nanoparticles, such as GO or magnetic particles to the furcellaran film, did not inhibit the growth of these microorganisms directly under the film. The obtained results were consistent with the data from literature, because CQDs exhibit bacteriostatic activity against *S. aureus* and *E. coli* [46,47].

The sulfate group in at C–4 and (1→4)-3,-anhydro-’α-D-galactopyranose caused the anionic nature of furcellaran [48]. The ζ-potential of furcellaran is −40 mV and ζ-potential γ-Fe_2_O_3_ nanoparticles are 33 mV [45,49]. The occurrence of the positively charged γ-Fe_2_O_3_ nanoparticles inclusion to the surface of anionic polysaccharides is known. The electrostatic attraction between the anionic sulphate groups (−SO_4_) on the furcellaran molecule and cationic patches (−Fe^2+^) on maghemite might interact and contribute to the lowering of antimicrobial effect of film. Similar types of electrostatic interactions between anionic polysaccharides and anionic proteins in aqueous solutions have been reported in the literature [50].

Based on the data from the literature, it can be stated that, along with the increase in inhibition zone of the antimicrobial component, its diffusion capacity also rises [51,52]. The obtained results indicate that selected films with the addition of nanoparticles can have an antimicrobial effect, without the diffusion of active ingredients into the environment. This effect would be highly desirable from the perspective of the possibility of using films as an active food packaging material. Nevertheless, further research should be conducted regarding the possibility of nanoparticle diffusion from furcellaran film into the external environment.

#### 3.3.2. Antioxidant Activity of Films

Some antioxidants are more effective as radical scavengers or lipid peroxidation inhibitors, while others have better metal chelating or reducing properties [53]. Therefore, the study involved assessing the antioxidant properties of samples while using three different in vitro tests: 2,2 diphenyl-1-picrylhydrazyl radical scavenging capacity (DPPH), iron ion reduction capacity (FRAP), and metal chelating capacity (Table 4).

The FRAP value for all types of films was low. The highest FRAP value was obtained for film with magnetic nanoparticles (0.010 mM/L) and the lowest for film with carbon quantum dots (0.002 mM/L). According to the data from literature [54], iron oxide nanoparticles, such as nanomaghemite, have demonstrated appreciable responses as antioxidants.

Measurements of antioxidant properties of furcellaran films via the DPPH method indicate that the addition of nanoparticles repeatedly increases their ability to scavenge free radicals. Films with the addition of graphene oxide and carbon quantum dots indicate the highest ability to scavenge DPPH radicals (11.69% and 10.29%, respectively). As reported by several studies focused on graphene oxide, functionalised carbon dots, fullerene, and carbon nanotubes, carbon nanomaterials may scavenge ROS [55,56]. According to this study, graphene oxide and carbon quantum dots showed significant antioxidant activity in the form of hydroxyl and superoxide radical scavenging.

The tested films with the addition of nanoparticles did not show the ability to chelate metal ions, except for the films with the addition of carbon quantum dots. Furcellaran film, with the addition of CQDs, showed the ability to chelate metal ions at a level of 18.63%. According to data from literature, CQDs’ chelating properties increase with an increasing concentration of this compound [57]. Further research is required to assess the effect of increasing the addition of CQDs to furcellaran films on their antioxidant activity.

### 3.4. Oil Stability after UV Treatment

During the normal exposure of oils to sunlight, oxidation mainly occurs due to exposure to UV. The UV radiation that causes oil oxidation is within the range of 280–380 nm due to the absorption of short-wavelength radiation by the Earth’s stratosphere and ozone layer [58]. Consequently, UV-B radiation is often used to accelerate the process of oil oxidation [58,59,60,61]. Although GO films do not show high absorption of UV light in the abovementioned spectrum, MAN and CQDs films have both exhibited UV-barrier properties (Figure 2). Therefore, a test for UV-induced oxidation of cold pressed flaxseed oil had been designed to study the efficiency of the UV-barrier properties. Table 5 shows the results of the oil stability analyses. 

The 30 min. UV-B treatment significantly increased the oxidation, as shown by the increase of PV and MDA content. Although an increase in FFE could also be observed, the differences were not statistically significant. Surprisingly, none of the films or the use of tinted glass inhibited MDA formation. Aside from light induced oxidation, the UV-B treatment resulted in a temperature increase of the oils, which were warm to the touch when removed from the UV chamber. This could be a possible drawback of the accelerated oxidation test, since even a mild temperature increase to 50–60 °C have been shown to affect the oxidation of oils, including MDA formation [62,63]. However, both MAN films and the use of tinted glass successfully inhibited the increase in PV. This might be due to the non-transparent brown color of MAN films. On the other hand, GO and CQDs films have not exhibited any protective effect. 

The degradation of carotenoids is an important factor in the oxidation stability of oils, since various carotenoids, such as β-carotene or lutein, have exhibited prooxidative properties after photoinduced oxidation of oils [64,65]. The content of carotenoids during oil stability tests significantly decreased after UV treatment. Surprisingly, the highest reduction was observed in MAN and tinted glass protected samples, which showed the best protective properties against the PV increase. On the other hand, GO films exhibited the best protective properties against the degradation of carotenoids. 

In the oil industry, the use of tinted glass bottles can be problematic for the manufacturer. Glass poses a potential physical hazard during the food manufacturing process and broken glass pieces are listed as one of the main physical hazards in hazard analysis by HACCP systems [66]. The use of glass bottles requires additional processing steps, such as bottle washing, sterilization and inspection [67]. Moreover, the glass bottles are susceptible to damage during transport, which results in additional potential hazards and waste of the product [68]. One possible solution is to use plastic bottles, however plastic bottles usually have lower consumer acceptance, are more permeable by gas and humidity, and can result in the formation of possibly toxic compounds [69]. Aside from problems with the use of glass, oil stored in tinted glass is less attractive to the consumer than oil that is stored in transparent bottles, due to lack of proper visibility of the product inside [70]. Consumers tend to assess not only the color of the stored oil, but also its turbidity, which is related to a product being more “natural” [71]. On the other hand, the use of transparent glass or plastic results in an increase in oxidation of the stored oil, resulting in lower quality and shorter shelf-life [70]. Therefore the transparent films, such as the GO and CQDs films presented in this study, can be used as an outer layer of a transparent glass or plastic bottle, thereby providing an additional barrier from UV and possibly from gas permeability while not compromising the transparency. However, the results of the oil stability test show that MAN was the only film that successfully inhibited oxidation, which was not transparent. On the other hand, during the oil stability test used in this study only one wavelength of 315 nm was tested and an additional test should be performed during which the oil should be exposed to natural sunlight for a prolonged period before discarding the described films from this particular application. 

## 4. Conclusions

Furcellaran films with three nanofillers: CQDs, MAN, and GO have been successfully developed, as shown through UV-Vis and FTIR analysis. The color and mechanical properties were affected by incorporating MAN and GO, but not by incorporating CQDs. Moreover, the films with CQDs exhibited radical scavenging and metal chelating properties. The films also exhibited antimicrobial properties in direct contact with microorganisms, but they did not affect the surrounding area, therefore suggesting that nanofillers were not released into the environment. CQDs and MAN films both showed UV-barrier properties, however, when subjected to an oil oxidation test, only MAN films showed a similar inhibitory effect, such as tinted glass. Future research directions will include the further use of nanocomposite films as packaging materials in biomedical applications.

## Figures and Tables

**Table 1:** The color of nanocomposite films.

Type of Films *	L *	a *	b *	ΔE
FUR	91.09 ^c^ ± 0.98	−0.49 ^a^ ± 0.04	5.73 ^a^ ± 0.13	
FUR + CQDs	91.76 ^c^ ± 0.37	−0.58 ^a^ ± 0.03	5.66 ^a^ ± 0.18	0.67
FUR + MAN	25.86 ^a^ ± 1.21	11.33 ^c^ ± 1.42	8.62 ^b^ ± 1.17	66.37
FUR + GO	82.62 ^b^ ± 0.75	0.50 ^b^ ± 0.08	11.06 ^c^ ± 0.59	10.06

* Values are expressed as mean ± standard deviation. Different letters in the same column indicate significant differences (*p* < 0.05).

**Table 2:** Effect of nanofillers on the physical properties of furcellaran films.

Type of Films *	Thickness [nm]	Tensile Strength [MPa]	Elongation at Break [%]	Water Content [%]	Solubility [%]
FUR	0.08 ^a^ ± 0.00	13.69 ^a^ ± 0.61	72.40 ^b^ ± 1.13	26.1 ^a^ ± 1.2	91.8 ^c^ ± 2.2
FUR + CQDs	0.08 ^a^ ± 0.00	13.71 ^a^ ± 0.69	72.26 ^ab^ ± 1.26	22.0 ^a^ ± 0.8	63.1 ^b^ ± 4.7
FUR + GO	0.08 ^a^ ± 0.00	18.66 ^b^ ± 0.05	71.00 ^ab^ ± 2.66	24.3 ^a^ ± 1.8	62.9 ^b^ ± 3.4
FUR + MAN	0.08 ^a^ ± 0.00	18.63 ^b^ ± 0.01	69.55 ^a^ ± 1.24	22.0 ^a^ ± 3.9	32.4 ^a^ ± 2.7

* Values are expressed as mean ± standard deviation. Different letters in the same column indicate significant differences (*p* < 0.05).

**Table 3:** The antimicrobial activity of nanocomposite films.

Type of Film	*S. aureus*	*E. coli*	*S. enteritidis*
FUR	-	-	+
FUR + CQDs	+	+	+
FUR + GO	-	-	+
FUR + MAN	-	-	-

Abbreviations: ‘-‘ no antimicrobial effect; ‘+’ inhibiting bacterial growth directly under the films; ‘++’ antimicrobial effect with inhibition zone.

**Table 4:** Antioxidant properties of the tested films.

Type of Films *	FRAP [mM/L]	DPPH [%]	Chelating Activity [%]
FUR	0.008 ^b^ ± 0.000	0.00 ^a^ ± 0.00	0.00 ^a^ ± 0.00
FUR + MAN	0.010 ^c^ ± 0.001	8.90 ^b^ ± 0.46	0.00 ^a^ ± 0.00
FUR + CQDs	0.002 ^a^ ± 0.000	10.30 ^bc^ ± 1.07	18.63 ^b^ ± 1.30
FUR + GO	0.005 ^b^ ± 0.001	11.69 ^c^ ± 1.76	0.00 ^a^ ± 0.00

* Values are expressed as mean ± standard deviation. Different letters in the same column indicate significant differences (*p* < 0.05).

**Table 5:** The oxidation parameters of flaxseed oil subjected to UV-B treatment.

Type of Sample *	PV [meq O_2_/kg]	FFA [mg KOH/g]	MDA [mmol/L]	Carotenoids [mg/kg]
Untreated	3.52 ^a^ ± 0.17	0.65 ^a^ ± 0.03	0.018 ^a^ ± 0.006	583.9 ^f^ ± 1.1
UV-Control	5.14 ^b^ ± 0.46	0.78 ^a^ ± 0.19	0.035 ^b^ ± 0.009	568.2 ^d^ ± 0.2
T	3.77 ^a^ ± 0.36	0.76 ^a^ ± 0.03	0.061 ^d^ ± 0.015	551.8 ^b^ ± 0.7
FUR + MAN	3.57 ^a^ ± 0.01	0.74 ^a^ ± 0.03	0.040 ^bc^ ± 0.005	525.7 ^a^ ± 0.9
FUR + GO	4.75 ^b^ ± 0.35	0.71 ^a^ ± 0.04	0.048 ^bcd^ ± 0.000	572.9 ^e^ ± 0.2
FUR + CQDs	5.03 ^b^ ± 0.42	0.71 ^a^ ± 0.04	0.055 ^cd^ ± 0.001	563.0 ^c^ ± 1.2

* Samples subjected to UV treatment using: tinted bottles (T), Petri dishes with maghemite films (FUR + MAN), Petri dishes with graphene oxide films (FUR + GO) and Petri dishes with carbon quantum dots films (FUR + CQDs). PV—peroxide value, FFA—free fatty acids, MDA—malondialdehyde, FRAP—ferric reducing ability of plasma. Results with different superscript letters (a-f) are statistically different (*p* < 0.05).
